# Concepts of advanced therapeutic delivery systems for the management of remodeling and inflammation in airway diseases

**DOI:** 10.4155/fmc-2021-0081

**Published:** 2022-01-12

**Authors:** Daljeet Singh Dhanjal, Parvarish Sharma, Meenu Mehta, Murtaza M Tambuwala, Parteek Prasher, Keshav R Paudel, Gang Liu, Shakti D Shukla, Philip M Hansbro, Dinesh Kumar Chellappan, Kamal Dua, Saurabh Satija

**Affiliations:** 1 School of Bioengineering & Biosciences, Lovely Professional University, Phagwara, Punjab, 144411, India; 2 School of Pharmaceutical Sciences, Lovely Professional University, Phagwara, Punjab, 144411, India; 3 Discipline of Pharmacy, Graduate School of Health, University of Technology Sydney, NSW, 2007, Australia; 4 School of Pharmacy & Pharmaceutical Sciences, Ulster University, Coleraine, County, Londonderry, BT52 1SA, Northern Ireland, UK; 5 Department of Chemistry, University of Petroleum & Energy Studies, Dehradun, 248007, India; 6 Centre for Inflammation, Centenary Institute & University of Technology Sydney, School of Life Sciences, Faculty of Science, Sydney, Australia; 7 Department of Life Sciences, School of Pharmacy, International Medical University, Bukit Jalil, 57000, Kuala Lumpur, Malaysia

**Keywords:** COPD, NDDS, remodeling, respiratory disorders

## Abstract

Chronic respiratory disorders affect millions of people worldwide. Pathophysiological changes to the normal airway wall structure, including changes in the composition and organization of its cellular and molecular constituents, are referred to as airway remodeling. The inadequacy of effective treatment strategies and scarcity of novel therapies available for the treatment and management of chronic respiratory diseases have given rise to a serious impediment in the clinical management of such diseases. The progress made in advanced drug delivery, has offered additional advantages to fight against the emerging complications of airway remodeling. This review aims to address the gaps in current knowledge about airway remodeling, the relationships between remodeling, inflammation, clinical phenotypes and the significance of using novel drug delivery methods.

Approximately 4 million premature deaths annually are reported due to chronic respiratory diseases (CRDs) worldwide [1], with ~339 million people suffering from asthma and 65 million people suffering from chronic obstructive pulmonary disease (COPD) [2]. Given this scenario, it is believed that there will be a substantial rise in the next decade, and COPD will become third leading cause of death in the world by 2030 [3,4]. Asthma is characterized by bronchial constriction, thickened and inflamed alveolar wall and airway remodeling. Symptoms include breathlessness, chest tightness, coughing, rhinitis and wheezing [5]. Generally, the occurrence and intensity of symptoms varies from person to person. The associated clinical symptoms relapse either spontaneously or during the treatment course. Additionally, the patient does not show any clinical symptoms of diseases during the diminution and reoccurrence of exacerbation of this disease [5,6]. COPD, in contrast, is associated with successive, partially irreversible airway constriction. This lung ailment is also linked to emphysema, pulmonary hypertension and chronic bronchitis. One causative factor of COPD is tobacco smoke. Further deterioration may also occur due to the chronic inflammatory response to numerous detrimental air pollutants and foreign particles [7,8].

The deterioration in the respiratory tract brings the radical change in the lungs, which is also known as ‘airway remodeling’ [9]. Remodeling predominantly concern the pathophysiology of the respiratory tract where structural transformation such as epithelial cell disruption, thickening of the base membrane due to collagen accumulation, excessive mucus secretion and cell infiltration by inflammatory cytokines obstructs the airway [10]. These successive variations in structural cells and tissues lead to the formation of a new airway wall with transformed physiology. These changes in the respiratory system hinder normal functioning of respiratory organs and prompt the demand for drugs to regulate these pathophysiological changes [7,9].

To curb the symptoms associated with these CRDs, various allopathic drugs, such as antihistamine, corticosteroids, bronchodilators, mast cell stabilizers and epinephrine are employed [11]. However, these therapeutic agents have associated side effects, including dyspepsia, dizziness, sour throat, xerostomia, rhinorrhoea and tremors. Although these therapeutic drugs are effective in managing CRDs, they require improvement in terms of extended drug release [12,13]. Furthermore, the barrier to reducing mortality is complex but could be linked to progress in the production of respiratory medicines, given the progress made in the past decade related to technology and healthcare. Many medicines do not pass the final stages of clinical trials; due to concerns about safety and effectiveness, only 3% of medications are eventually marketed, resulting in long manufacturing cycles of 15–20 years and expenditure of more than USD 1.5 billion per approved medicinal product [14,15]. The factors that contribute to this are multifactorial and complex. One factor is the challenge of current methods of modeling human physiology and disease with static noncomplex *in vitro* and animal modeling. Inadequate models coupled with methods that are not physiologically appropriate for the deposition of respiratory medicines indicates the need for a more specific physiological model of human lungs and methods of delivery [16,17]. Within this context, the current review focuses on the anatomy and cellular physiology of the respiratory system, provides insight on airway remodeling and inflammation response associated with CRDs such as asthma and COPD. It also discusses the challenges faced during the drug development for respiratory diseases and highlights the potential and new outlooks of novel drug delivery systems to treat CRDs.

## Anatomy & cellular physiology of human lungs

**Figure 1.**
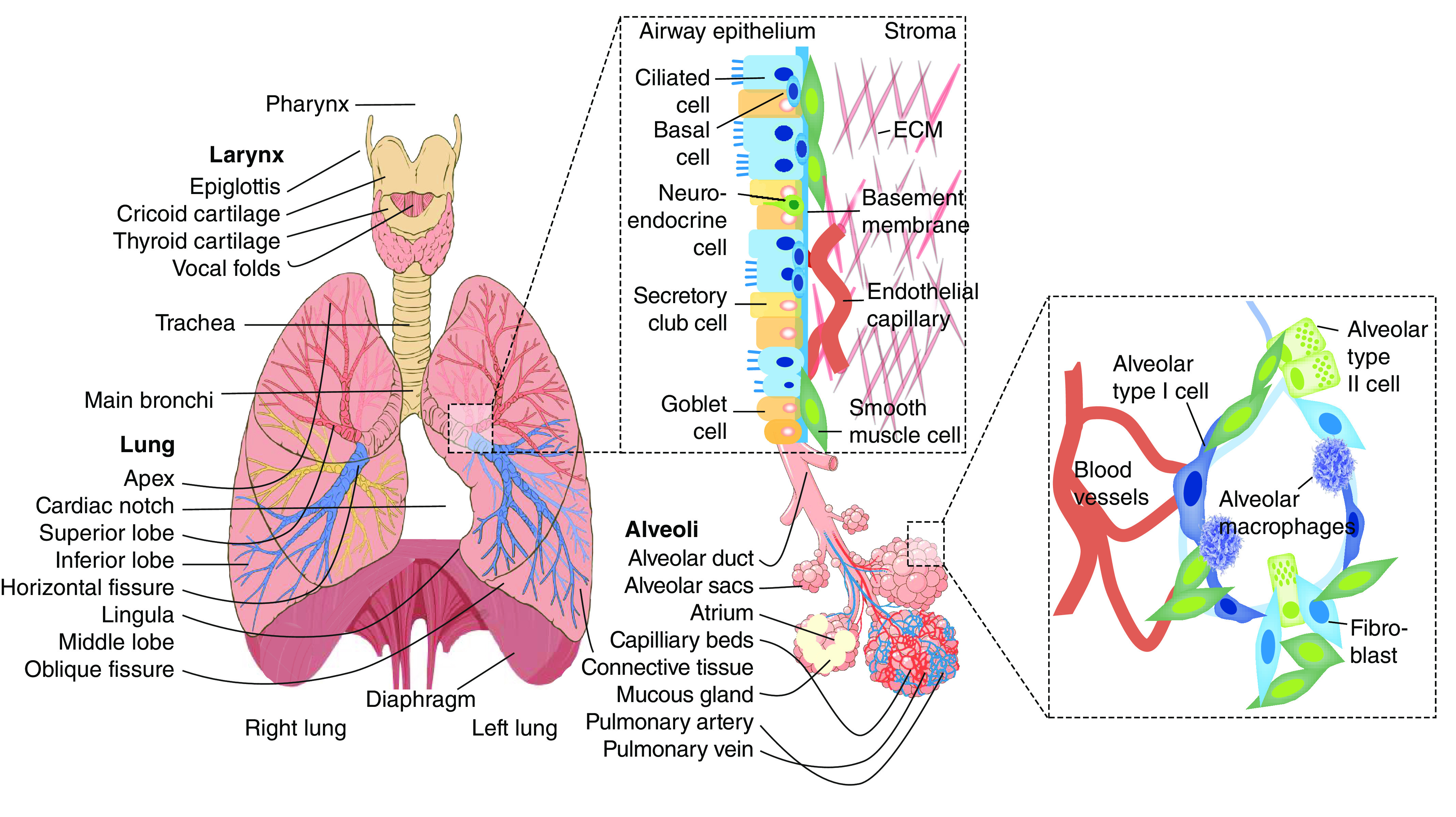
Anatomy of respiratory system and airway epithelium.

The lung is the paired organ that performs the elementary function of gas exchange – that is, removal of carbon dioxide (CO_2_) from deoxygenated blood and transfer of oxygen (O_2_) to the blood from inspired air [18]. The lung is divided in two parts (Figure 1); the left lung contains two lobes the and right lung contains three lobes. Interestingly, the airways are ~1500 miles long, encompassing 350–500 million alveoli to perform the function of gaseous exchange. The matured lungs have a surface area of ~70 m^2^ [19,20]. In fact, if we unwound the capillary vessels that surround the alveoli and place them in a straight line, it would extend to 600 miles. Inhaled air is transferred to the lungs via trachea, bronchi and bronchioles. These complex conducting airways are not involved in gas exchange but, along with cartilage, provide strength to maintain apparency for exhalation and inhalation of air [21,22].

The conducting zone contains cells with small projections known as cilia, which clean the inhaled air via wavelike motions. The cilia propel a foreign entity back to the large bronchi, from where it reaches the trachea, and the cough reflex then completes the expulsion process of the entity. Additionally, immune and mucus-secreting cells align along the airway and help entrap bacteria, dust, pollen, viruses and foreign entities to protect the lungs [23,24]. Exchange of gases predominantly takes place in the respiratory system, which involves bronchioles, alveolar ducts and alveoli. The epithelial layer of the alveoli contains two main types of cells – that is, type I pneumocytes (covering 95% surface of alveoli) and granular type II pneumocytes (responsible for secreting pulmonary surfactant and repairing impaired alveoli). Type I pneumocytes intersect with the lining of pulmonary capillaries (made up of endothelial cells) to enable the exchange of CO_2_ and O_2_ through the alveolar–capillary membrane [25,26].

The maximum lung capacity varies from 4 to 6 l depending on age, gender, weight and height. The lung capacity of females is 25% less than males. Characteristically, normal respiratory rate is 15–20 breathes in 60 s, and for each breath ~500 ml of air is exchanged [27]. Despite this, lungs have remarkable reserve volume because under a forced condition, it can inhale and exhale 4 l of air. In reality, the average human breathes ~11,000 l of air daily, which contains 2.31 l of oxygen [28].

## Airway remodeling

**Figure 2.**
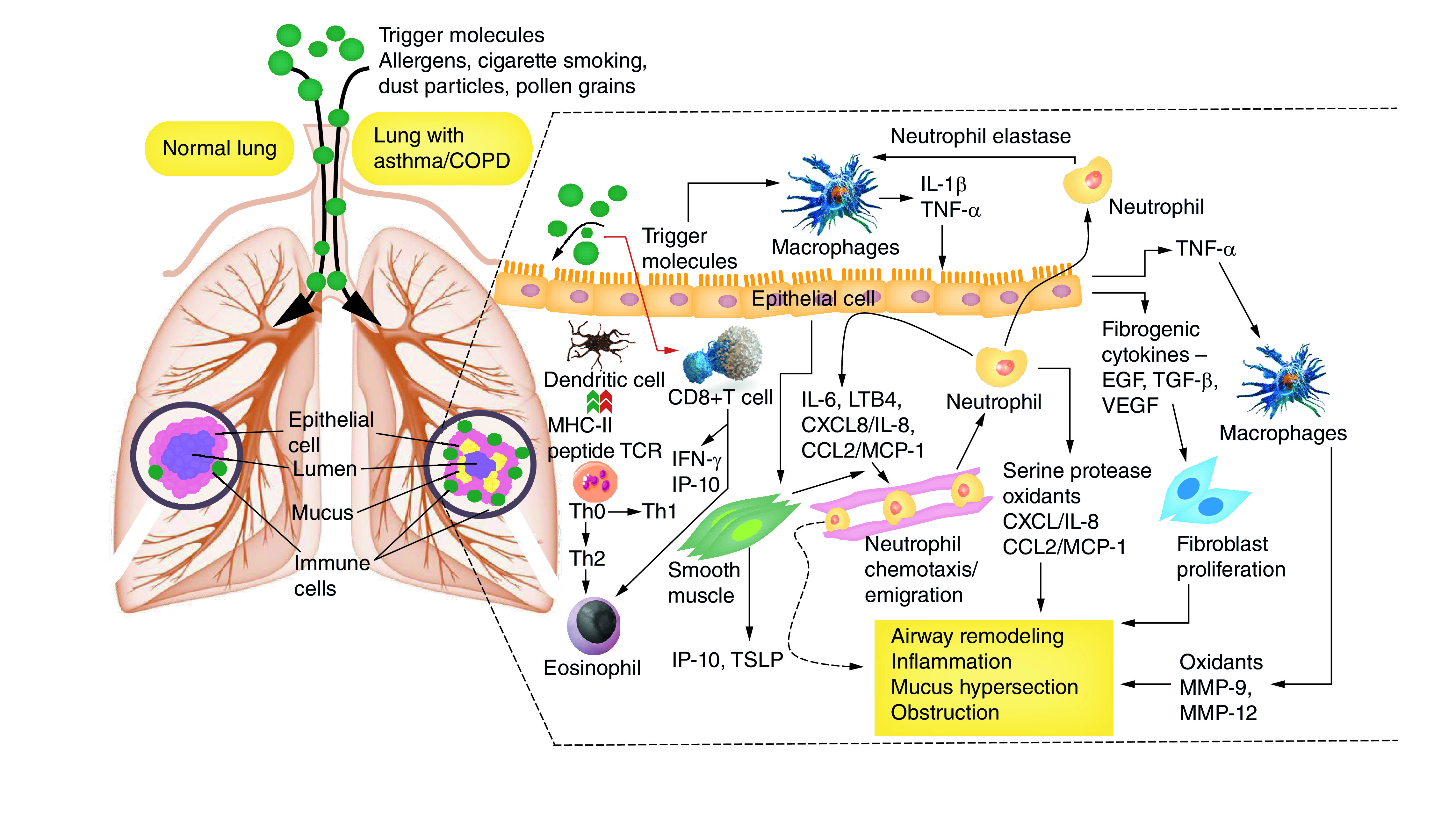
Different structural changes and inflammation response in the lung (with asthma and chronic obstructive pulmonary disease). COPD: Chronic obstructive pulmonary disease.

Asthma and COPD are the chief respiratory disease, which are a chronic inflammatory response in conducting airway and the lung parenchyma [29,30]. Inflammation in the airways causes hindrance in the airflow and stimulates the inflammation mediator to directly act on airway smooth muscles (ASMs). The persistent inflammation leads to structural changes such as an increase in the number of ASMs and fibrosis of the airway wall [31]. These structural changes cause thickening of the airway wall, leading to narrowing of airway and obstruction in airflow [32]. In both asthma and COPD, structural and cellular changes known as remodeling obstructs maintenance of normal functioning and the morphology of airways and parenchyma of lungs [9,33] (Figure 2).

### Structural changes

**Figure 3.**
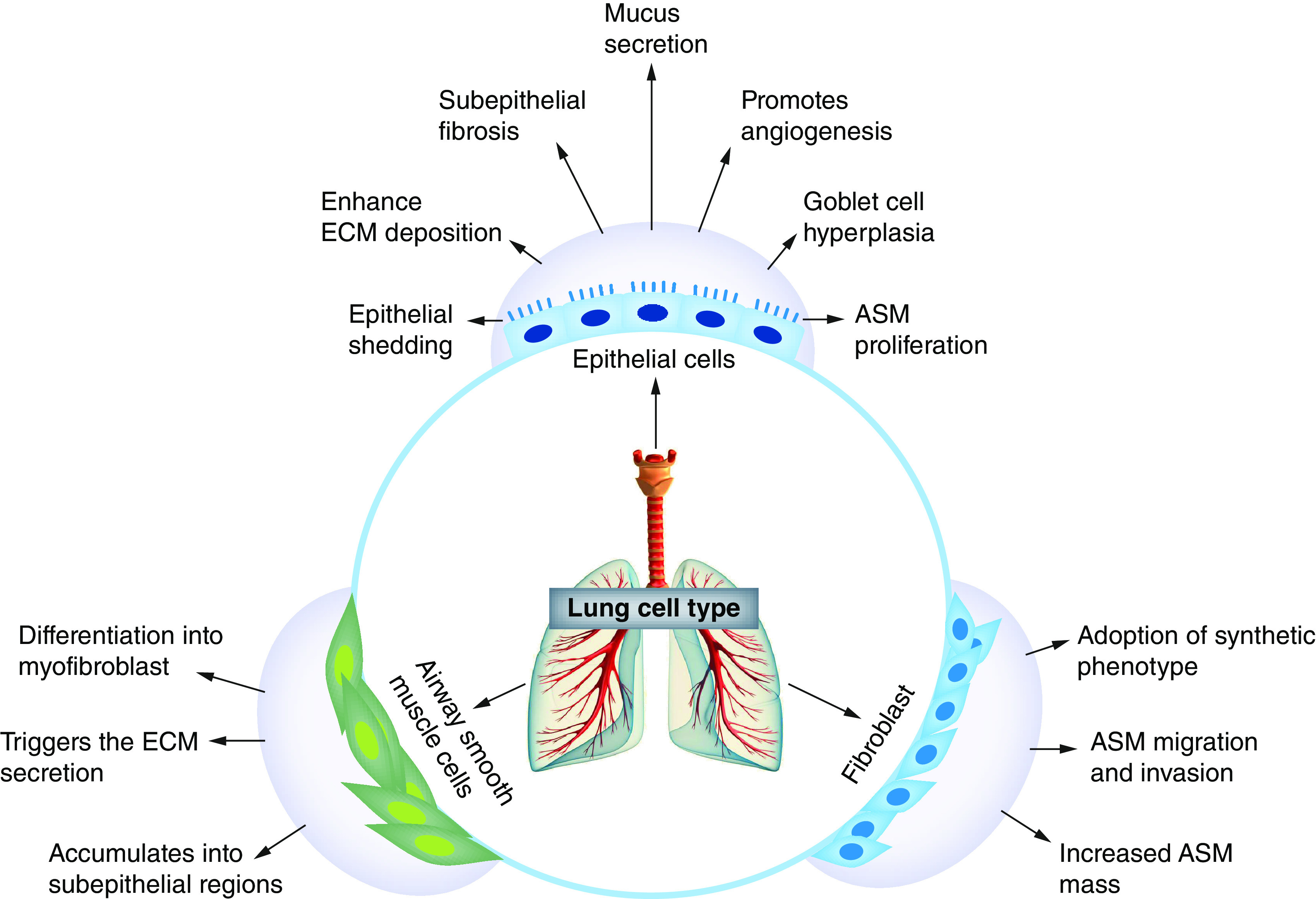
Contribution of different types of cells in pathology during airway remodeling in respiratory diseases. ASM: Airway smooth muscle; ECM: Extracellular matrix.

One of the characteristic features persistent in both asthma and COPD is the constricted airflow experienced during the transition from acute to chronic inflammation and remodeling of the airway wall and lung parenchyma [34,35]. In asthma, airway remodeling is characterized by structural changes involving an increase in the number of ASMs, epithelial damage, thickening of the subepithelial reticular basement membrane (RBM), mucous gland hypertrophy, vascular congestion and subepithelial fibrosis [7,36]. Additionally, airway structural changes can occur in the absence of chronic inflammation [37], as evidenced by some studies in children. According to a study conducted by Bossley *et al.*, severely asthmatic children aged 6–16 years showed markers of eosinophilia and remodeling in the absence of TH2 cytokines [38]. A biopsy investigation in preschool children with severe wheeze reported the existence of various remodeling markers, including enhanced RBM thickness, increase in the number of ASMs, vascularity and mucus gland hypertrophy; however, there is no link with inflammatory biopsy cell counts and remodeling [39]. These are structural changes that are responsible for the thickening of the airway wall and obstructed airflow. Characteristic features of airway remodeling in COPD are different from asthma because it involves subepithelial fibrosis, hypertrophy of the mucous gland and metaplasia of goblet and squamous cells (Figure 3) [40,41]. These structural changes not only cause airflow constriction, but the increased liberation of inflammatory and mucous exudate also increases surface tension, which is involved in airway closure [42]. Hypertrophy/hyperplasia conditions lead to an increase in the number of ASM cells, which accounts for the narrowing of the airway wall and reduces the airflow in severely asthmatic patient [43]. It has also been noted that fibromyocytes containing α-actin differ from myofibroblasts at the ultrastructural level. These cells are dedifferentiated myocytes that migrate themselves to subregional part of large airway and transform themselves to new ASM bundles. In contrast, aggregates of ASMs take place in smaller instead of larger airways [43,44].

In asthma, wall edema and vascular congestion are two important components that are involved in airway thickening, whereas medial hypertrophy and intimal thickening are observed in pulmonary artery in case of COPD [45]. Interestingly, infiltration of T lymphocytes, especially the CD8+ phenotype in pulmonary arteries, has been noted in the literature on COPD. Shedding airway epithelium is recorded in asthmatic patients, whereas goblet and squamous cell metaplasia are observed in COPD patients [46,47].

Remarkably, the mitotic potential of airway epithelium that plays a critical role in the restoration of the uncovered area is suppressed in asthma but not COPD. This explains the abnormal restoration response of epithelium cells during injury [48,49]. Defective repair or chronic injury of airway epithelial cells results in activation and secretion of several growth factors and proinflammatory epithelial-derived cytokines such as EGF, eotaxin, RANTES, GM-CSF and IL-8 [50–52]. Further, continuous release of these growth factors and cytokines cause and perpetuate changes in structural morphology in asthma, which involves the hypertrophy of mucous glands and ASMs and RBM thickening. Thickening of RBM is a characteristic feature that is observed in asthma but not COPD. However, fibrosis of peripheral cells of the airway is observed in COPD [53,54]. In addition, inflammatory alterations such as hypersecretion of mucus, airway edema, damage of alveolar walls and loss of elastic recoil are a few factors associated with narrowing of the airway in COPD [55]. Further, augmentation of extracellular connective tissue matrix, myofibroblasts and fibroblasts in COPD is mediated via numerous cell types, such as epithelial cells and macrophages, which synthesize fibronectin, PDGF, TGF-β and IGF-1. Data related to the fibrosis of the airway wall in asthma remains unclear [56,57].

Recently, functional and pathological characteristics of patients suffering from constricted airflow due to asthma and COPD are under study. Patients with COPD have a high percentage of neutrophils in bronchoalveolar lavage (BAL) fluids and induced sputum; in contrast, asthmatic patients have a high percentage of eosinophils in BAL fluid, airway mucosa, blood and sputum. These are significant clinical and pathological characteristics that separate these CRDs [58,59].

### Inflammation

Eosinophils have a significant association with airway hyperresponsiveness and asthma. Eosinophils are the prominent and abundant inflammatory cells, along with CD4^+^ T lymphocytes, mast cells, macrophages and neutrophils, found in the airway of the asthmatic patient [58,60]. Eosinophils infiltrate the entire respiratory system, but their distribution varies in both small and large airways. The predominance of eosinophils in small airways is observed in the outer part of the airway wall, in contrast to larger airways, where eosinophils are extensively observed in the inner part of the wall (i.e., between the basement membrane and smooth muscle) [49,61,62].

Inflammation due to neutrophils is also observed in asthmatic patients. However, airway inflammation in COPD patients is different from asthma because it involves infiltration with neutrophils, T lymphocytes and macrophages [63]. The lumen airways show the predominance of macrophages and neutrophils, confirmed from findings in which their elevated levels were recorded in BAL fluid and sputum [64,65]. Additionally, prominence of CD8+ T lymphocytes is observed in the airways of COPD patients in contrast to CD4^+^ T lymphocytes in asthma patients. The correlation of increased CD8+ T lymphocytes with the complication of airflow obstruction supports the theory that these lymphocytes have a role in inflammation response during the development of COPD. Although the key role of CD8+ T cells is unknown, they synthesize perforin and granzyme, which are responsible for damage to tissue [66,67]. The literature has also revealed that VEGF regulates the immune and inflammation response in both asthma and COPD [68]. Further, extensive research has been conducted on the inflammatory cells involved in activation and recruitment processes that are regulated by various cytokines. The expression profiles of cytokines are found to be distinct for asthma and COPD [69,70]. Eosinophils and T-helper (Th2) T lymphocytes are abundant in airway inflammation, and different cytokines are found to regulate the various inflammation process in asthmatic patients [71]. Such cytokines are categorized as Th2-like cytokines (IL-4, -5, -9, -13), growth factors EGP and TGF-β, chemokines (eotaxin, MCP-1 and RANTES) and proinflammatory cytokines (IL-1β and TNF-α), whereas neutrophil chemokines and proinflammatory cytokines play a vital role in the case of COPD [71,72]. BAL fluids, biopsy and sputum of COPD patients show the increasing level of IL-1β/6/8, TNF-α and MCP-1. In acute cases, the airway of COPD patients shows increased levels of eosinophil and neutrophils, along with the elevated expression of cytokines such as CXCL5/8, eotaxin and RANTES [73].

## Challenges in respiratory drug development

**Figure 4.**
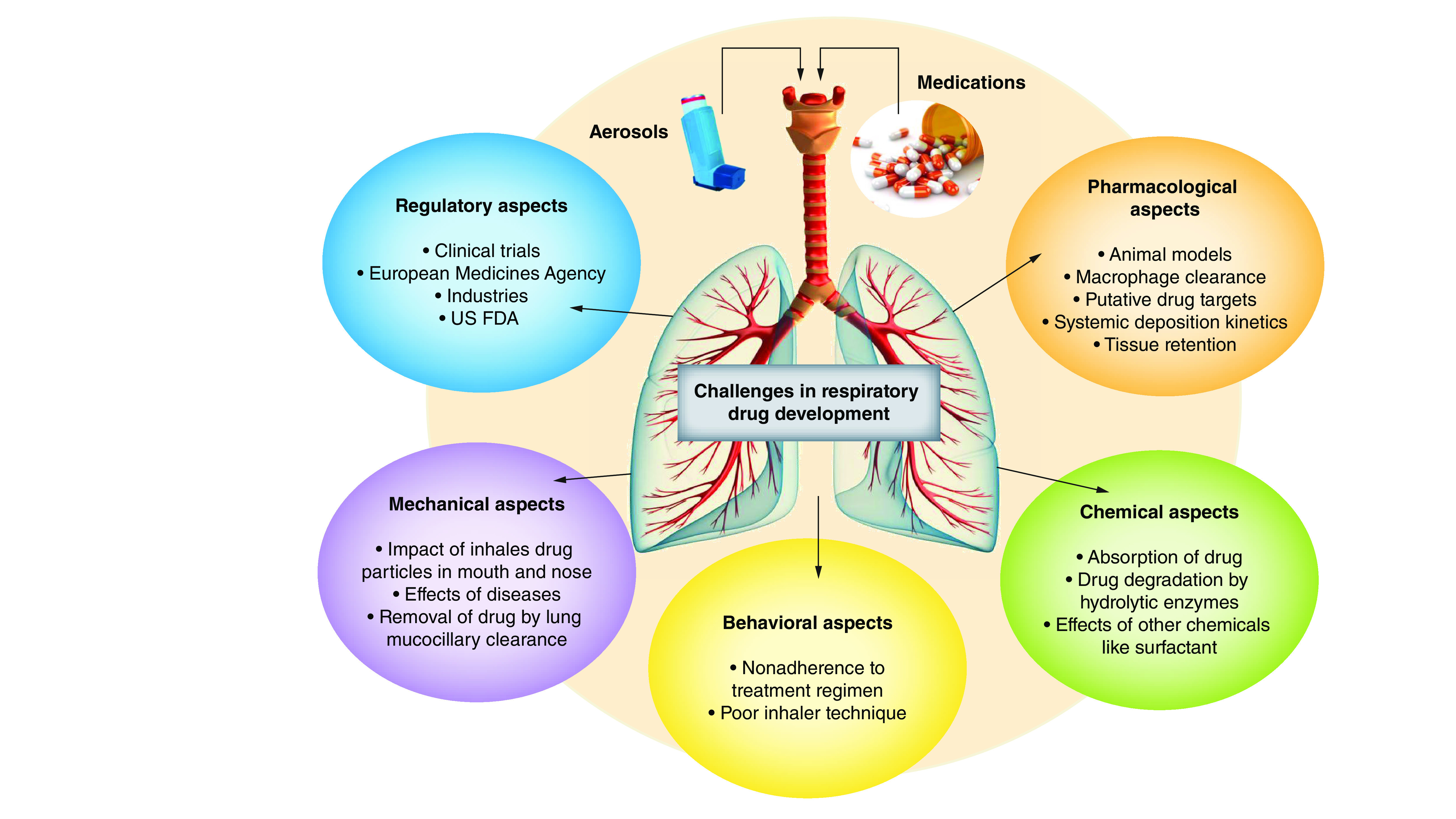
Challenges involved during respiratory drug development.

Despite various unmet medical needs in respiratory medicine, limited effective and safe therapies have been developed in the past 40 years. Following are some of the challenges associated with drug development of these respiratory diseases (see also Figure 4).

### Behavioral challenges

#### Adherence

Drug delivery in pulmonary diseases is significantly influenced by patients’ use of their inhaling device. Adherence indicates the number of dosages taken relative to the number of prescribed dosages [74,75]. Nonadherence to these therapeutic routines is common and may be either intentional or unintentional because when the patient feels healthy, decides not follow the prescribed regimen or just simply forgets to use it. ‘True adherence’ relates to both regimen adherence and proper inhaler technique, and it is now thought that optimizing true adherence is important for future disease management [76]. Misconceptions and cultural factors also play critical roles in adherence level [77,78]. For example, a survey conducted in India stated that there is social stigma related to carrying inhaler, although people believed that inhalers are used for treating chronic diseases [79,80].

#### Inhaler approach

Insignificant inhaling technique has long been documented as a limitation of inhaled drug delivery, and a recent published review stated that patients over the past 4 decades, patients have continued not to know how to use inhalers properly [81]. The chief mistake during inhaler use for pressurized metered dose inhalers (pMDI) involves not activating the inhalers while breathing in and being unable to inhale slowly and deeply [82,83]. The major problem with dry powder inhaler (DPI) involves incompetent inhaling with adequate force, along with preparation and device handling errors, mainly due to improper orientation if the device. The majority of patients forcefully inhale the DPI, but many elder patients lack the muscle strength required for proper DPI use [84,85]. Inadequate breath-hold rest after inhalation and being unable to exhale before inhaling are some of the challenges associated with both the types of inhalers. Improper assembly of nebulizer equipment and holding the face mask away from the face are other problems with inhaling technique [86,87]. Moreover, behavioral, chemical, mechanical and immunological barriers also need be taken into consideration to ensure effective delivery of the different types of drugs at the targeted site [88,89].

### Chemical & immunological barriers

During normal lung functioning, various entities enter and are deposited in the respiratory system. These particles are thought to dissolve within the lung fluids, but how this process works remains the unknown. Drugs are not eliminated by mucociliary clearance, but theoretically they either exert their effect on nearby tissues or are absorbed during systemic circulation [90,91]. These deposited drugs may be subjected to chemicals such as surfactants and proteases (proteolytic enzyme). Proteolytic enzymes such as cathepsin H and neutral endopeptidase hydrolyses the proteins as well as peptides causes the inactivation of these drugs in the lungs [92]. Some of the undissolved particles are also phagocytized by alveolar macrophages, which act as an immunological barrier because they do not distinguish between potentially beneficial substances and harmful ones [93,94]. Therefore, these macrophages can engulf the drug molecules and eliminate them from the lungs through the lymph system. The role of macrophages in drug absorption has been studied in animal models but is still not well understood in humans. In the case of surfactants, they restrict the adhesion of drug particles on the lung surface and make drug particles more accessible to macrophages [95,96].

### Mechanical barriers

The lungs comprise a complex network of branching airways referred as the bronchial tree [97]. To reach the large epithelial cells in the alveolar region, a particle must pass through multiple bifurcation airways, where it may be deposited. The recommended aerosol size should have aerodynamic diameter less than ~5 μm for delivery of drugs in the lungs [98]. To deliver the drug to the alveolar epithelium, the particle size should be even smaller than aerodynamic diameter, approximately 3 μm [99,100]. Furthermore, deposition of drug particles depends on inhalation parameters such as breath-hold pause, inhaled volume and inhale flow rate. During pMDI drug delivery, the inhaled flow rate should be slow, whereas for DPI drug delivery, the inhaled flow rate should be forceful and fast. Approximately 20% of the inhaled dose reaches the lung, while majority of drug is deposited in the oropharynx (for DPIs and pMDIs). Mechanical barriers impose great challenges in respiratory diseases because the airways are narrowed due to bronchoconstriction, inflammation and hypersecretion of mucus [101–103].

### Pharmacological challenges

The high failure rate of respiratory drugs during the drug discovery process has made it difficult and expensive to introduce new drugs or chemical therapeutic entities to the market. For every approved drug, more than 10,000 chemical entities are rejected at each stage of development. These limitations have prompted researchers to attempt to improve the efficacy of drug-related research and development [104,105]. Given the demand for therapeutics, pharmaceutical companies have to speed up the production of new chemical entities each year to maintain their status and profits, but after observing the current scenario, it seems an impossible task. For instance, of the drugs selected for phase I clinical trials from 1991 to 2000, only 11% reached for the registration of phase II and III clinical trial studies [106,107]. However, after registration, approximately 25% of drugs also fails to clear clinical trials due to the cost involved in development. The cause of drug failure has been extensively studied to reduce the massive waste of pharmaceuticals. Before 2000, bioavailability and poor pharmacokinetics were the chief cause of drug failure. Therefore, pharmaceutical companies have started investing in the application and development of highly precise and accurate modeling and prediction approaches to substantially reduce the chance of failure [108].

Lack of efficiency is the most common cause of drug failure and is particularly common in respiratory diseases because animal models are unable to mimic human conditions in preclinical and clinical trials. For example, during 2011–2012 ~60% of drugs failed due to lack of efficacy. It is believed that early proof of concept studies can improve the drug discovery process and reduce the cost involved in drug development. Additionally, a common cause of attrition is safety and toxicity issues, with ~33% drugs failing for this reason during phase III clinical trials [109,110]. This is apparently a great risk for small chemical entities with novel mechanism of action, but there is less risk of failure for toxicological reason because the compounds will be eliminated on assessment of the mechanism-based toxicity. Furthermore, improvement of toxicity models also reduces the chances of failure. Hence, there is need to determine biomarkers to predict the efficacy of a drug, which could serve as a key element for improving the move from drug discovery to development of valuable new clinical treatments [98].

### Regulatory challenges

Recently, collaboration among academia and pharmaceutical industries has led to various scientific breakthroughs in understanding cellular, mechanistic and molecular physiology of respiratory diseases. This has prompted us to develop novel therapies for patients who lack proper medical treatments [111,112]. According to a literature survey on innovative medicines for the period 2010–2013, of 141 medicines, only eight were selected by the US FDA and EMA in accordance with standardized procedures for respiratory drugs. This selection percentage is low with regard to social, scientific and marketing relevance to respiratory diseases [113,114]. Of those eight, only four ultimately went to market: which involves roflumilast for COPD, pirfenidone for idiopathic pulmonary fibrosis, ivacaftor for cystic fibrosis and riociguat for idiopathic pulmonary hypertension [115,116]. It is noteworthy that marketing authorization does not mean that a drug will be readily available in the market. This implies that assimilation of new medicine in the market is difficult, slow and risky. Even after the marketing authorization, drug pricing and health technology assessment (HTA) at the national level obstructs patient access to medicines [117,118]. This process will become more difficult in the future as the cost of drugs are significantly increasing. Additionally, nonavailability of clinical trial data as a source of information for research in academia and the pharmaceutical industry also delays the availability of drugs on the market. Hence, it has become important for the WHO to take an initiative in the collaboration of regulatory bodies worldwide to facilitate early access to innovative drugs [119–121].

## Novel drug delivery for human airway remodeling

### Nanoparticles

**Figure 5.**
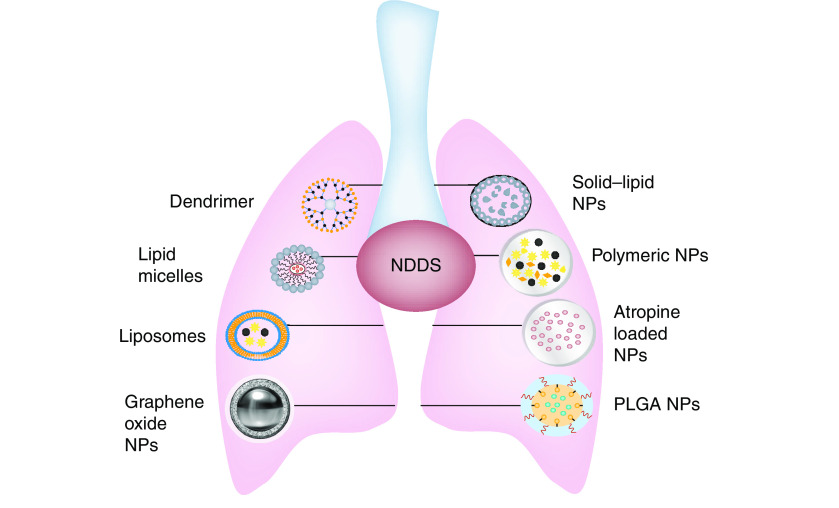
Nanocarriers for drug delivery in the lungs. NP: Nanoparticle; PLGA: Poly(lactic-co-glycolic acid).

Nanoparticles (NPs) have become the most extensively studied class of drug delivery systems, and publications discussing nano drug delivery are exponentially increasing [122–125]. Due to the wide range of capabilities and unique properties of these NPs, there has been substantial improvement in conventional drug delivery systems [126–129]. Therefore, efforts are being made to develop new and advanced nano drug delivery systems to improve patience compliance, enhanced efficacy and optimal treatment safety [130]. For effective and safe drug delivery to the lungs, NPs must be resistant to various changes in lung parenchyma during the progression of lung disease as well as to external stimuli (change in pH, activation of neutrophils, complement system and macrophages, heat, clearance of subepithelial fibrosis and excessive mucus production) [64,131–133]. Currently, nanomedicine has made significant progress in developing targeted NPs for particular organs [134]. However, inside the organs, diseases show spatial heterogeneity because distinct pathological and healthy regions can be observed. Hence, successive steps are being taken to target the specific region affected in the disease and treat it [135]. With this aim, a range of nanocarrier systems have been developed as promising strategies for treating the ailments involved in airway remodeling (Figure 5).

The synergism between lung fibrosis and COPD is involved in inflammatory response, which results in the activation of macrophages, interleukins and proteases such as MMP-9 and MMP-12 [136]. Airway remodeling is the type of fibrosis that occurs in asthma. Extracellular matrix (ECM) deposition and proliferation of ASM cells around the airway can result in narrowing of the airways, airflow obstruction and dyspnea [137]. The biocompatible nature of both organic and inorganic NPs makes them tolerable for various organs, especially the lungs. Thus, both these organic and inorganic NPs are recommended for targeted delivery in pulmonary system because they do not induce inflammation. Both NPs have the additional advantage of being compatible with lipophilic drugs. Moreover, these nanocarriers have low toxicity and improved drug stability [138–140].

Au and TiO_2_ NPs have been suggested as effective NPs in treating COPD [131]. Another study reported on immune-conjugated poly(lactic-co-glycolic acid) (PLGA) NP formulation for targeted delivery of ibuprofen to neutrophils in COPD murine model [141]. Graphene oxide (GO) NPs were tested for their ability to modulate allergic pulmonary response in an asthmatic murine model. This study revealed that these NPs decrease eosinophil accumulation and increase the number of macrophages in BAL fluid. Additionally, the presence of GO also stimulated the production of ovalbumin-specific IgG2a, acid mammalian chitinase (AMCase) and CHI3LI and downregulated the production of IgG1 and IgE. These changes led to potentiation of hyperresponsiveness and airway remodeling [142].

Juan *et al.* synthesized ATG101 antisense RNA-loaded DNA triangular NPs (ssATG101-TNP) to suppress ATG101 gene expression, which is important in pulmonary vascular remodeling. They demonstrated that ssATG101-TNP can be effectively transported to human pulmonary arterial endothelial cells in a time- and dose-dependent manner. Decreased expression of ATG101 promotes cell apoptosis while inhibiting hypoxic cell autophagy and proliferation, making it a potential therapeutic target for endothelial injury-related conditions [143]. This is further supported from the study conducted by Veronica *et al*., who tested the efficacy of targeted imatinib-loaded gold nanoparticles in patients with systemic sclerosis interstitial lung disease and in a lung fibrosis experimental model. Gold NPs were prepared using anti-CD44 and loaded with imatinib. Results showed that these NPs significantly reduced lung fibrotic changes and collagen deposition in a bleomycin lung fibrosis mouse model. These NPs inhibit proliferation of lung fibroblasts and significantly decrease the release of IL-8 in alveolar macrophages [144].

A highly compacted DNA nanoparticle produced from poly-lysin and polyethylene glycol copolymers can trigger the release of thymulin analog genes and significantly decrease the inflammatory and remodeling process in a mouse model of allergic airway diseases [145]. Moreover, Yoo *et al.* developed PLGA-based polymeric nanoparticles containing antiinflammatory compound (hydroxybenzyl-alcohol-conjugated polyoxalate), which was administered in an ovalbumin-induced asthma mice model intratracheally. The result obtained from the study showed reduction in the level of proinflammatory cytokines [146].

Nassimi *et al.* have highlighted the application of triglyceride- and phospholipid-based solid lipid nanoparticles (SLNs) at a ratio of 70:30 for treating pulmonary diseases. They conducted a study to assess toxicity of these SLNs *in vivo*, *in vitro* and *ex vivo* along with cytokine activation measurements. The results showed no activation of proinflammatory cytokines [147,148].

Chattopadhyay *et al.* formulated biodegradable atropine-loaded NPs (ANPs) to assess the hyperresponsiveness of lungs on their administration in adult Wistar rats. Their results showed inhibition of inflammatory cytokines, normalized tidal volume of obstructive and hyperresponsive lung and reduced shallow breath. After 18-day 18 days with ANPs, a reduction in collagen deposition and improvement in progressive airway obstruction were noted [149].

### Carbon nanotubes

Carbon nanotubes (CNTs) are the nanomaterials that structurally resemble asbestos due to their fiber-like nature and biopersistence [150]. Li *et al.* have developed an animal model and investigated the negative impact of single-walled CNTs on lung function symptoms through detection of airway hyperresponsiveness. Single-walled CNTs exacerbated histological changes in lung tissues in the concentration ranges from 0.20 and 2.00 mg·kg^-1^ compared with the control. Histopathological analysis clearly showed the presence of airway remodeling, deposition of collagen, mucus production and hyperplasia [151]. Another study showed the role of STAT1 in murine allergen-induced airway remodeling and exacerbation by CNTs. Another study showed that STAT1 is an essential factor in protecting the fibrogenic reaction to multiwalled CNTs by reducing development of TGF-β2, intracellular Smad2/3 phosphorylation and collagen synthesis [152]. The presence of cellular debris and mucus in the airways of diseased patients changes the surface charge, surface area and aggregation status of CNTs. Moreover, people suffering from asthma or COPD are reported to have impaired mucociliary clearance, which further obstructs the effectiveness of CNT clearance compared with healthy individuals [153].

Advanced properties of nanomaterials can offer tremendous benefits, but they can also endanger human health and the environment. Most of the nanomaterials are in the size range of particulate matter, so nanomaterial exposure can have harmful effects on the lungs. Many industries use nickel NPs as catalysts to produce multiwalled CNTs. Baker *et al.* investigated the role of Tbx21 in lung remodeling by nickel NPs. Results showed that nickel NPs increase the mucous cell metaplasia, chronic alveolitis and IL-13 count in T-bet deficient mice. One study concluded that nickel NPs in T-bet-deficient mice with prior allergic pulmonary inflammation worsened airway remodeling [154]. It is known that IL-13 is a key mediator in allergic asthma. The involvement of this cytokine in the progression of asthma and COPD makes it the target cytokine for both diseases. Thus, anti-IL-13 therapies are being developed as an effective solution to regulate the clinical manifestation of asthma and COPD [155,156]. Intrinsically, CNTs are insoluble in aqueous solutions and organic solvents. Moreover, their easy amendment with chemicals enhances their biocompatibility, transforming these CNTs into water-soluble nanocarriers and reducing toxicity. CNTs have a large surface area, and their cavity can hold high payloads. The unique electron emission, mechanical and optical properties make it a candidate of interest. Moreover, their high penetration power and functionalized surface provide additional benefit to aid in targeted delivery [157].

### Polymeric micelles

Polymeric micelles, which are self-assembling nano-constructs of amphiphilic copolymers with a central core, have been used as dynamic carriers for drug and nucleic acid delivery [158]. They offer various advantages, such as the ability to solubilize poorly soluble drugs, biocompatibility, stability and the ability to accumulate in pathological areas with compromised vasculature [158]. Budesonide-containing polymeric micelles have been developed by Sahib *et al.* and assessed for reducing inflammatory cell counts in the bronchoalveolar region in a COPD/asthma rat model [159]. Another study showed that budesonide-containing micelles reach into the deeper areas of the lung due to their small particle size and their sustained release, which prolongs the antiinflammatory effect for up to 24 h [160].

Mammalian chitinases have been investigated as a significant factor in the development of fibrosis and airway remodeling [161]. Chil 3 and Chil 4 are rodent-specific acidic mammalian chitinases reported as candidate protein biomarkers for allergic asthma. As demonstrated by Choi *et al.*, siRNA/HMG/OR micelle ternary complexes could be used to deliver Chil3 and Chil4 siRNA and Toll-like receptors (TLR4+) in alveolar macrophages and the bronchial epithelium can be targeted with these complexes. Their findings indicate that the pulmonary inflammation reduced by the downregulation of Chil3 or Chil4 in alveolar macrophages. This inflammatory attenuation was accompanied by a reduction in the production of mucus and airway remodeling [162].

Human mesenchymal stromal cells (MSCs) are helpful in reducing inflammation and tissue remodeling. Considering this as important biological process, De Castro *et al.* assessed the therapeutic potential of extracellular vesicles derived from human adipose tissues MSCs on airway remodeling in an experimental allergic asthma model. Results showed that these extracellular vesicles modulate airway remodeling and also that these vesicles reduced the level of proinflammatory markers, eosinophil count and collagen fiber content in lung tissue [163]. The polymeric micelles have emerged as potential nanocarriers because they are composed of hydrophobic copolymer core that enables pay-loading of hydrophobic chemotherapeutic agents, whereas their hydrophilic shell allows the payload of hydrophilic chemotherapeutic agents. Additionally, the hydrophilic shell improves the stability of these polymeric micelles. The micelle size is in range of 20–100 nm, making it a candidate of interest or therapeutic drug delivery by accommodating high drug permeability, high payload, uniform distribution and long circulation time in blood. Today, the polymeric micelles are being used for personalized treatment, and this approach has gained significant attention for its application in targeted drug delivery [164,165].

In addition, dendrimer (bifurcated and branched polymeric macromolecules) formulations have been developed to treat endothelium dysfunction and prevent inflammation in pulmonary diseases [166]. Currently, Arikace (amikacin, Insmed, NJ, USA) and Pulmaquin (ciprofloxacin, Aradigm Corp., CA, USA) liposomal products are in their last stage of clinical trials developed for treating diverse lung diseases [167,168]. These nanocarriers are fabricated chemically with a controlled polymeric reaction involving an hydrophobic and electrostatic interaction. Mostly, these dendrimers are found in globular form and have a functional group attached on their surface that makes them a candidate for targeted drug delivery [169,170]. Another group of researchers formulated phospholipid-based liposomes with the help of antioxidants such as glutathione, n-acetylcysteine and vitamin E using a film method. The result of an *in vivo* study revealed the reduction in proinflammatory cytokine levels in the BAL fluid in a rat model [171,172]. The main reason to use liposomes for targeted delivery is their unique features (e.g., nontoxic nature, physical stability, high retention time at the targeted site, high vascular density and easy amendment of the surface by external stimuli). Thus, different types of liposome formulation are being developed for targeted delivery of therapeutic agents [33]. The combinations of the various approaches contribute in one way or another to the research and development of drugs for regeneration of airway tissues.

## Conclusion & future perspective

Airway remodeling in respiratory diseases is a multicellular process that results in structural changes that contribute to pathogenic mechanisms, bronchoconstriction and sometimes a lack of response to treatment. The complexity of human airway linings makes the production of new therapeutic moieties difficult because traditional methods fail for numerous reasons. New therapeutic approaches based on stimulating the regenerative potential of the lung itself are needed as a means to treat airway disease. Understanding the various pathways and targeted provision of drugs to start, maintain, modulate and achieve normal lung growth may be necessary for new approaches to regeneration through the reactivation of the pulmonary pathways. Many drugs are being studied for their ability to delay remodeling along with antiinflammatory and antiapoptotic effects; however, they must be compared with currently used front-line treatments and analyzed for their possible use as an additional treatment with corticosteroids and beta-agonists. Given that remodeling exacerbates respiratory illness, translational, clinical and drug delivery scientists are exploring an evolving and challenging area of study, particularly in the complex and uncertain times of a global pandemic. Advances in drug delivery, especially NPs, are providing a competitive advantage in mitigating the incipient challenges of airway remodeling in chronic respiratory diseases. It is our hope that novel drug delivery system and new interventions could provide effective therapies to treat asthma and COPD.

**Executive summary**

**Airway remodeling**

- Chronic respiratory diseases are caused primarily by transformation in the airways of respiratory tract, a process known as airway remodeling.
- Reduced smooth muscle mass due to airway fibrosis, mucous metaplasia and glandular hypertrophy are other features associated with these chronic disorders.

**Inflammation**

- TGF-β1, CTGF and PDGF play important roles in airway remodeling and epithelial damage, which promote inflammation and fibrosis.

**Challenges in respiratory drug development**

- Although there are many unmet medical needs in respiratory medicine, only a few effective and safe drugs have been developed in the past 40 years.

**Novel drug delivery for human airway remodeling**

- The use of novel drug delivery systems for pulmonary delivery of drugs offering an extra advantage in the fight against airway remodeling issues in chronic respiratory disorders.
